# *Ziziphus jujuba* (Rhamnaceae) Alleviates Working Memory Impairment and Restores Neurochemical Alterations in the Prefrontal Cortex of D-Galactose-Treated Rats

**DOI:** 10.1155/2021/6610864

**Published:** 2021-05-31

**Authors:** Antoine K. Kandeda, Danide Nguedia, Espoir R. Ayissi, Jonas Kouamouo, Théophile Dimo

**Affiliations:** ^1^Department of Animal Biology and Physiology, Faculty of Science, University of Yaoundé I, P.O. Box 812, Yaoundé, Cameroon; ^2^Department of Pharmacy, University of the Mountains, P.O. Box 208, Banganté, Cameroon; ^3^Department of Biological Sciences, Higher Teacher's Training College, University of Yaoundé I, P.O. Box 47, Yaoundé, Cameroon

## Abstract

Alzheimer's disease is a progressive cognitive dysfunction. However, pharmacological treatments are symptomatic and have many side effects, opening the opportunity to alternative medicine. This study investigated the antiamnesic effect of the aqueous extract of *Ziziphus jujuba* on D-galactose-induced working memory impairment in rats. Impairment of working memory was induced by subcutaneous (s.c.) injection of D-galactose (350 mg/kg/day) to rats for 21 days. These animals were then subjected to object recognition and Y-maze tests. Rats with confirmed memory impairment were treated *per os* (*p.o.*) with tacrine (10 mg/kg), aspirin (20 mg/kg, *p.o.*), extract (41.5, 83, and 166 mg/kg, *p.o.*), and distilled water (10 mL/kg, *p.o.*) daily for 14 days. At the end of the treatments, alteration in working memory was assessed using the above paradigms. Afterward, these animals were euthanized, and cholinergic, proinflammatory, and neuronal damage markers were analyzed in the prefrontal cortex. Rats administered D-galactose and treated with distilled water had impaired working memory (evidenced by decreased time spent on the novel object and discrimination index) and decreased spontaneous alternation in the Y-maze. D-galactose also decreased the levels of acetylcholinesterase and acetylcholine and increased the level of glial fibrillary acidic protein, ionized calcium-binding adapter molecule 1, tumor necrosis factor-alpha (TNF-*α*), interleukin 1 beta (IL-1*β*), interleukin 6 (IL-6), and interferon-gamma (IFN-*γ*). Treatment with the extract (166 mg/kg) reversed the time spent on the novel object and the discrimination index. It equally increased the percentage of spontaneous alternation. Neurochemical analysis revealed that the extract markedly alleviated acetylcholinesterase activity and neuroinflammation. These observations were corroborated by the reduction in neuronal loss. Taken together, these results suggest that *Ziziphus jujuba* aqueous extract possesses an antiamnesic effect. This effect seems to involve cholinergic and anti-inflammatory modulations. This, therefore, claims using this plant in the treatment of dementia in Cameroon subject to further studies and trials.

## 1. Introduction

Alzheimer's disease (AD) is the most common form of dementia in the aging population. This condition is associated with progressive degeneration of thinking and memory [1]. This condition affects the cerebral cortex and hippocampus and leads to progressive degeneration in brain function.

AD is estimated to affect over 44 million people worldwide [2, 3]. According to the WHO, the number of people affected by this disease is expected to double every 20 years, from 65.7 million in 2030 to 115.4 million in 2050 [4, 5]. The risk factors for AD include aging, hypertension, obesity, stroke, obesity, and diabetes. Despite the enormous research on AD and its etiology, the exact molecular mechanisms underlying AD remain unclear [6]. Nonetheless, research in animals and humans suggests that the central degeneration characteristic of AD can be classified into three categories: neurofibrillary pathology, deposits of amyloprotein beta peptide, and loss of cholinergic neurons in the hippocampus [7]. Furthermore, AD is linked in part to a deficiency in the brain neurotransmitter, acetylcholine, and the inhibition of acetylcholinesterase (AchE) is important for the treatment of AD. Several scientific findings implicate other pathways such as oxidative stress, excitotoxicity, apoptosis, and neuroinflammation in the pathophysiology of AD [8]. This latter can be modeled in rodents by the systemic administration of certain chemicals such as scopolamine or D-galactose. D-galactose is a monosaccharide sugar found in dairy products, avocados, sugar beets, and mucilages; it is used as a model for senescence in age-related neurodegenerative diseases [9]. Accumulating evidence has shown that chronic systemic exposure of rodents to D-galactose leads to progressive neuronal loss and associated memory impairment [6]. These effects appear to be mediated through oxidative stress, neuroinflammation, among others and converge to lead to neurodegeneration [10]. To manage AD, many drugs are currently being used (Tacrine, Donepezil, Rivastigmine, Galanthamine, and Memantine) [11]. Unfortunately, these treatments are symptomatic and have many side effects including insomnia, anorexia, diarrhea, fatigue, nausea, gastrointestinal disorders, and cardiovascular disorders [12]. The plant products on the other have long been used in treating memory impairment. *Ginkgo biloba*, *Catharanthus roseus*, *Bacopa monnieri*, *Acorus calamus*, and *Centella asiatica* are some of the plants whose extracts are used in the herbal formulation as remedies for AD. Therefore, herbal remedies could potentially be a great source for the discovery of effective and safe drugs against AD. According to the WHO, herbal medicine has become a major component of primary health care [13]. *Ziziphus jujuba*, which is the subject of the present study, is a plant of the Rhamnaceae family, used in Asia and African traditional medicines to treat asthma, sleep disorders, obesity, gastrointestinal disorders, urinary disorders, and neurodegenerative diseases [14, 15]. In Cameroon, according to the claims of traditional healers, the fruits are used to treat ear infections, rickets, and anorexia [16, 17]. Also, the seeds are used as dewormers, while the leaves are used to treat mental disorders [16, 17]. In recent years, *Z. jujuba* has been shown to exert nephroprotective [18], hepatoprotective [18], anxiolytic, antioxidant, and sedative effects [19, 20]. The fruit of *Z. jujuba* was shown to possess anti-inflammatory [21] and neuroprotective [22] activities. Besides, the leaves of *Z. jujuba* were shown to possess anti-inflammatory and antioxidant properties [23, 24]. Neuropharmacological studies showed that the ethanolic extract of the seeds improves cognitive dysfunction in experimental models of AD in rats [25, 26] and ameliorates seizures and cognition in experimental models of seizures in rats [27]. Phytochemical analysis by high-performance liquid chromatography indicates the presence of (‒)-catechin, quercetin-3-*O*-robinobioside, rutin, chlorogenic acid, caffeic acid, ferulic acid, ellagic acid, epicatechin, and quercetin-3-*O*-*α*-*L*-arabinosyl-(1⟶2)-*α*-*L*-rhamnoside in the leaves of *Z. jujuba* [28–30]. These abundant bioactive compounds may account for the pharmacological properties of the plant [30–32]. The ethanolic extract of *Z. jujuba* has been shown nontoxic [22, 24]. Despite the interesting reports on the beneficial effects in ameliorating several disorders as highlighted, there is, currently, no report on the antiamnesic effect of the aqueous extract of *Z. jujuba.* Therefore, this study aimed to examine the antiamnesic effect of the aqueous extract of *Z. jujuba* on memory impairment induced by D-galactose in rats. The involvement of cholinergic and anti-inflammatory pathways was also explored.

## 2. Materials and Methods

### 2.1. Plant Material and Extract Preparation

The leaves of *Z. jujuba* used in this study were harvested in Mora (Far-North region, Cameroon), in November 2018. The whole plant was identified at the National Herbarium of Cameroon (Yaoundé) by Mr. Ngansop Tchatchouang Eric and compared to sample N°14446/HNC. To prepare the aqueous extract, the healer macerates two teaspoons (50 g) of leaf powder in 5 L of tap water for 72 h. Then, he filters the mixture and prescribes a full glass six-time daily to an adult of about 70 kg. This corresponds to a daily intake of approximately 3.6 L. In our laboratory, the extract of Z*. jujuba* was prepared mimicking the traditional healer's method. Briefly, the leaves of *Z. jujuba* were washed, dried in the shade, and crushed. The resulting powder was macerated (50 g) in 5000 mL of distilled water for 72 h. In the end, the mixture was filtered using Whatman number 3, and the macerate (stock solution) was evaporated in an oven at 45°C. A stock solution was made from the dry extract (6 g), representing a 12% yield. Thus, the dose administered to an adult of about 70 kg was 13.45 mg/kg. To obtain the corresponding dose in rats, the dose above was multiplied by 6.17 (metabolism factor), giving an 83 mg/kg dose. Also, to achieve a dose-dependent effect, the stock solution was either diluted two times to a dose of 41.5 mg/kg and concentrated 2 times to a dose of 166 mg/kg. Therefore, solutions were administered *per os* (*p.o.*) to rats at doses of 41.5, 83, or 166 mg/kg.

### 2.2. Animals and Ethics

3-month-old male rats (Wistar), weighing between 150 and 250 g, were used for this study. These animals were acquired from a private animal house and acclimatized for 7 days at the Laboratory of Animal Physiology (University of Yaoundé I) and were kept 4 per cage (50 cm × 50 cm) under ambient temperature (26–28°C) and natural light/dark cycle. During the experiments, the rats had free access to food and tap water *ad libitum*. The study was carried out following the principles governing the use of laboratory animals by the national (No: FWA-IRB00001954) and international (NIH publication No: 8023, revised 1996) ethic committees. All efforts were made to reduce the number of animals used.

### 2.3. Chemicals

Tacrine, acetylsalicylic acid (aspirin), D-galactose, and other chemicals or reagents were purchased from Sigma Chemical laboratories, St. Louis (USA), while ethyl ether was purchased from Cooper laboratory (France). The doses of aspirin (20 mg/kg) [31, 32] and tacrine (10 mg/kg) [33, 34] were determined based on previous laboratory findings and dose-response curve [35].

### 2.4. Methods

#### 2.4.1. Experimental Design and Induction of Memory Loss with D-Galactose

The experimental rats were randomly divided into two groups as follows:

- Group I (*n* = 7): rats received subcutaneously (s.c.) distilled water (10 mL/kg)
- Group II (*n* = 52): rats received D-galactose (350 mg/kg, s.c.)

Rats in groups above were administered the corresponding substances once daily for 21 days. At the end of the treatments, the animals that received D-galactose were subjected to object recognition and Y-maze tests. Rats with memory loss were selected and divided into 6 groups of 7 rats each as follows:

- D-galactose group received distilled water (10 mL/kg, *p.o.*)
- Test groups received Z. *jujuba* extract (41.5, 83, or 166 mg/kg, *p.o.*)
- Positive control groups received tacrine (10 mg/kg, *p.o.*) or aspirin (20 mg/kg, *p.o.*)

**Figure 1:**
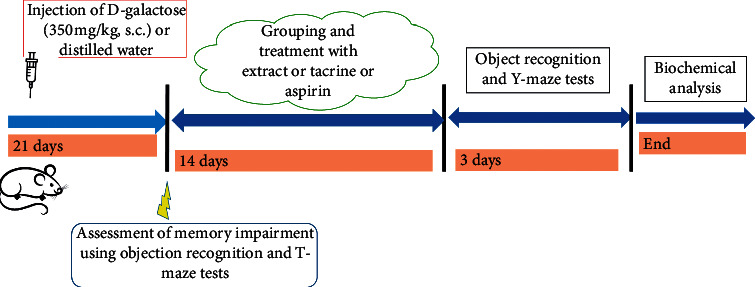
Schematic diagram of the experimental procedure. s.c.: subcutaneously.

The seventh group of mice (normal group) received distilled water (10 mL/kg, *p.o.*). These groups were treated once daily for 14 days. Three hours following the last treatment, the rats were subjected to object recognition and Y-maze tests. Afterward, they were euthanized and the prefrontal cortex was collected for the analysis of cholinergic and proinflammatory markers. Histological analysis of the prefrontal cortex was performed elsewhere (Figure 1).

#### 2.4.2. Object Recognition Test

The object recognition test was performed as previously described [36]. This test has three phases: habituation, acquisition, and retention. The first phase of the test was devoted to habituation. In this phase, rats were individually familiarized with the open field for 5 min, to reduce stress due to neophobia. The second phase of the test or acquisition phase began 24 h after the habituation session. In this assay, which also lasted 5 min, the rat was placed in the presence of two identical objects (A + A) for free exploration. A retention trial took place 24 h after the acquisition phase. This phase was carried out in the same way as the acquisition phase, except that one of the objects (A) was replaced with a new object (B). The exploration time of each of the two objects (A and B) was recorded as tA and tB, respectively. A rat is exploring a particular object when it points its muzzle at the object at least 2 cm away from the object. When the rat sits on or walks around the object, this is not considered an exploration. A discrimination index (DI) was defined as the time spent exploring the new object (B) divided by the total exploration time of the two objects [36]. DI = tB/(tB + tA), where tA is the exploration time of object A and tB is the exploration time of object B.

#### 2.4.3. Y-Maze Test

The Y-maze test was used to assess working memory in animals by recording spontaneous alternation [37]. The maze used was a wooden device with 3 identical branches called arms (40 cm long × 35 cm high × 12 cm wide) which were separated by an angle of 120°, and the walls of each arm were decorated with a different pattern (A, B, and C) to differentiate them. Rats were individually placed at the end of a branch of the maze to freely explore the maze for 5 min [38], and the number of entries in each arm of the maze was recorded. After each animal session, the device was cleaned with 10% ethanol. Spontaneous alternation (SA) is defined as three successive entries in the three different arms (example: ABC, CAB, or BCA). The percentage of SA was used as an index of performance of the working memory and calculated according to the following formula [37]: [(number of SA)/(total number of arms visited  − 2)]^*∗*^100.

#### 2.4.4. Animal Euthanasia and Preparation of Homogenates

Following the behavioral analysis, animals were euthanized by decapitation after inhalation of ethyl ether for 2 min. Brains were collected as fast as possible, washed in 0.9% NaCl, wrung out, and placed into boxes containing frozen 0.9% NaCl. Frozen organs were dissected to extract the prefrontal cortex (*n* = 5). On the one hand, one-half of the tissues were used to prepare homogenates (20%) with Tris buffer (50 mM HCl; 150 mM KCl; pH 7.4). The homogenate was centrifuged at 3000 rpm for 25 min, and the supernatant was stored at −20°C. The second half of the brain (*n* = 2) was fixed in 10% formaldehyde for histological analysis. The relative weight of the prefrontal cortex, expressed in percentage, was determined by the following formula: [(weight of the prefrontal cortex (mg)/weight of the brain (mg)]^*∗*^100.

#### 2.4.5. Determination of Acetylcholinesterase Activity

Dinitrothiocyanobenzene (DNTB) reacts with acetylcholinesterase (AchE) to form a yellow-colored complex that absorbs at 412 nm [39, 40]. The amount of AchE was estimated by Ellman's method [41]. For the estimation of AchE activity, 20 *μ*L of 0.1 M Tris-HCl buffer (pH 8.0) and 3 mL of the Ellman reagent were added to all tubes followed by 100 *μ*L of homogenate or 100 *μ*L of Tris buffer (50 mM HCl; 150 mM KCl; pH 7.4) in the blank tube. Subsequently, 20 *μ*L of 30 mM acetylthiocholine iodide was added to all tubes. The mixture was read at 412 nm at times 30 s and 90 s after mixing. The absorbance of all samples was read against the blank. The enzymatic activity was expressed in *μ*mol of hydrolyzed iodide acetylthiocholine iodide/mg of tissue/min.

#### 2.4.6. Determination of Acetylcholine Level

The acetylcholine (Ach) content was estimated using Hestrin's method [42]. Briefly, tissue was placed in a test tube, which was boiled for 10 min to denature the activity of AchE. The tissue was then homogenized in 2 mL of distilled water. To homogenate, 1 mL of alkaline hydroxylamine hydrochloride was added, followed by 1 mL of HCl (0.1 M). The contents were carefully mixed and centrifuged. To the obtained supernatant, 0.5 mL of 0.37 M ferric chloride was added, and after the development of brown color, the solution was read at 540 nm against the blank. The acetylcholine content was expressed in *μ*mol of acetylcholine/g of tissue.

#### 2.4.7. Determination of Glial Cells and Proinflammatory Cytokine Marker Levels

The quantification of glial fibrillar acid protein (GFAP), ionized calcium-binding adapter molecule-1 (Iba-1), interleukin 1 beta (IL-1*β*), interleukin 6 (IL-6), tumor necrosis factor-alpha (TNF-*α*), and interferon-gamma (INF-*γ*) was carried out by the enzyme-linked immunosorbent assay (ELISA). Quantikine ELISA kits (Biotechne, Inc., Minneapolis, USA) were used according to the supplier's instructions.

#### 2.4.8. Histopathological Analysis of the Prefrontal Cortex

The histological analysis included fixing, cutting, dehydration, inclusion, cutting, coloring, mounting, and observation. The stained and mounted slides were observed at ×250 magnification using Scientico STM-50 optical microscope (HSIDC Industrial Estate, Haryana, India). This apparatus was equipped with a Celestron 44421 digital camera connected to a computer.

#### 2.4.9. Phytochemical Screening

The presence of coumarins, anthraquinones, anthocyanins, alkaloids, tannins, sugars, phenolic compounds, flavonoids, carboxylic compounds, saponins, triterpenes, and sterols was ascertained according to Bruneton's method [43].

#### 2.4.10. Quantitative Phytochemical Screening

All quantitative phytochemical assays were performed in triplicate.

*(1) Total Phenolic Content*. Total phenol level was assessed using the Folin and Ciocalteu (FC) method [44]. The concentrations (0.02–0.15 mg/mL) of standard (gallic acid) and plant extract were prepared with the reaction mixture containing 100 *μ*L of gallic acid, 500 *μ*L of FC reagent, and 400 *μ*L of 7.5% sodium carbonate. The mixture was then incubated at room temperature for 10 min, and the absorbance was read at 730 nm by a spectrophotometer. The concentration of total phenol compounds in gallic acid equivalent was determined from the calibration curve of gallic acid and expressed in mg gallic acid equivalent (GAE)/g of plant extract. *(2) Total Flavonoid Content*. The estimation of total flavonoid content was carried out by the method of Dehpour et al. [45]. Different concentrations (0.02–0.15 mg/mL) of standard (rutin) and plant extract were prepared with 500 *μ*L rutin added to 1500 *μ*l of 95% (v/v) methanol, 100 *μ*L of 10% (m/v) aluminium chloride, 100 *μ*L of 1M sodium acetate, and 2.8 mL of distilled water. The mixture was stirred and incubated in the dark at room temperature for 30 min. The absorbance was measured at 415 nm, using a spectrophotometer. Results were determined from the calibration curve of rutin and expressed in mg rutin equivalent (RE)/g of plant extract [46]. *(3) Condensed Tannins*. Condensed tannins content was determined by the vanillin hydrochloride method [47]. The reagent was prepared by mixing an equal volume of 8% (v/v) hydrochloric acid, 37% (v/v) methanol, and 4% vanillin in methanol (m/v). The mixture was kept at 30°C before the assay. 200 *μ*L of plant extract or catechin at different concentrations (0.06–0.3 mg/mL) was added to 1000 *μ*L of vanillin reagent mixture and incubated in the dark for 20 min at 30°C. The absorbance was read at 500 nm by a spectrophotometer. The total of condensed tannins in catechin was calculated from the catechin calibration curve and expressed in mg catechin equivalent (Cat E)/g of plant extract [46]. *(4) Determination of Total Alkaloids*. Five grams of the plant extract was weighed into a 250 mL beaker and 200 mL of 10% acetic acid in ethanol was added, and all was covered and allowed to stand for 4 h. The mixture was filtered and the obtained extract was concentrated on a water bath to one-quarter of the original volume. Concentrated ammonium hydroxide was added drop-wise to the extract until the precipitation was complete. The whole solution was allowed to settle, and the precipitate was collected, washed with dilute ammonium hydroxide, and filtered. The residue was the alkaloid, which was dried and weighed [48]. The level of total alkaloids in the plant extract was determined as follows: % alkaloids = [weight of alkaloids (mg)/weight of the plant extract (g)]^*∗*^ 100. *(5) Determination of Total Saponins*. The plant extract (20 g) was put into a conical flask and 100 cm^3^ of 20% aqueous ethanol was added. The solution was heated over a hot water bath for 4 h with continuous stirring at about 55°C. The mixture was then filtered and the residue was reextracted with another 200 mL ethanol (20%). The concentrate was transferred into a 250 mL separatory funnel, and 20 mL of diethyl ether was added and shaken vigorously. The aqueous layer was recovered while the ether layer was discarded. The purification process was repeated and 60 mL of n-butanol was added. The combined n-butanol extracts were washed twice with 10 mL of 5% aqueous sodium chloride. The remaining solution was heated in a water bath; and after evaporation in the oven at 55°C, a dried extract was obtained and the total saponin concentration was calculated as follows [48]: % saponins = [weight of saponins (mg)/weight of plant extract (g)]^*∗*^100.

### 2.5. Statistical Analysis

Statistical analysis of the values obtained was carried out using GraphPad Prism version 8.0 (San Diego, CA, USA) and Microsoft Office Excel 2013 version 15.0.4420.1017. Results were expressed as mean ± standard error of the mean (SEM) or as a percentage. Data were first assessed for normality and sphericity using Shapiro-Wilks and Mauchly's tests, respectively. These analyses confirmed that repeated measures of data were normally distributed, but some data violated the assumption of sphericity, in which the Greenhouse-Geisser, Benjamini, and Krieger and Yuketieli corrections were performed [49]. One-way ANOVA followed by Tukey's *post hoc* test was used to analyze data from the number of spontaneous alternations in the Y-maze test, discrimination index in the object recognition test, and Ach, AchE, and proinflammatory cytokines levels in the prefrontal cortex. Repeated two-way ANOVA followed by Bonferroni Multiple Comparison *post hoc* test was used to analyze exploration time in the object recognition test. For data that showed a nonnormal distribution like GFAP level, Iba-1 level, and relative weight of the prefrontal cortex, the Kruskal-Wallis test was used. Regression analysis was used to assess the correlation between the number of entries in the arms and the percentage of spontaneous alternation in the Y-maze. At *P*<0.05, the difference was considered significant.

## 3. Results

### 3.1. Effect of *Z. jujuba* Extract in the Object Recognition Test

**Figure 2:**
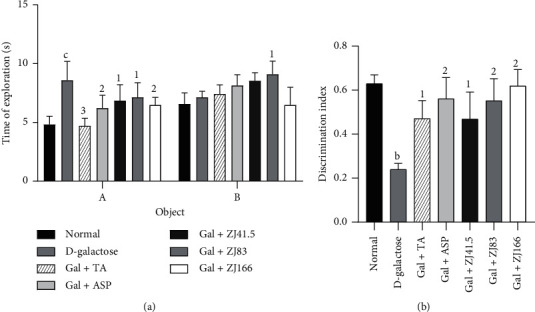
Effect of *Z. jujuba* extract on object recognition. (a) Time of object exploration. (b) Discrimination index. Each value represents the mean ± SEM; *n* = 7; b*P*<0.01, c*P*<0.001: significant difference compared to the normal group; 1*P*<0.05, 2*P*<0.01, 3*P*<0.001: significant difference compared to D-galactose group; DW: distilled water; Gal: D-galactose; ASP: aspirin; TA: tacrine; ZJ: *Ziziphus jujuba*; Normal: normal group only treated with distilled water; D-galactose: negative control group treated with D-galactose and distilled water; Gal + TA: positive control group treated with D-galactose and tacrine (10 mg/kg); Gal + ASP: positive control treated with D-galactose and aspirin (20 mg/kg); Gal + ZJ (41.5, 83, and 166): test groups treated with D-galactose and the aqueous extract of *Z. jujuba* at the respective doses of 41.5, 83, and 166 mg/kg.

Injection of D-galactose to the D-galactose group increased (*P*<0.001) the time for exploring the old object (A), while it decreased this of the new object (B) when compared to the normal group (Figure 2(a)). The extract at the dose of 83 mg/kg increased (*P*<0.05) the exploration time of object B, while it reduced (*P*<0.05) the time for exploring object A at the dose of 166 mg/kg. Both tacrine and aspirin reduced (*P*<0.05) the time for exploring object A, while they failed to significantly increase the time for exploring object B (Figure 2(a)).

The evaluation of the discrimination index showed that this value increased to 0.24 ± 0.03 (*P*<0.01) in the negative control group when compared to the normal group (Figure 2(b)). The extract (83 and 166 mg/kg) showed the greatest effect by increasing this index to 57.80% (*P*<0.01) and 61.90% (*P*<0.01), respectively. Tacrine and aspirin increased this parameter to 39.66% (*P*<0.01) and 58.76% (*P*<0.01), respectively (Figure 2(b)).

### 3.2. Effect of Z. jujuba Extract in the Y-Maze Test

**Figure 3:**
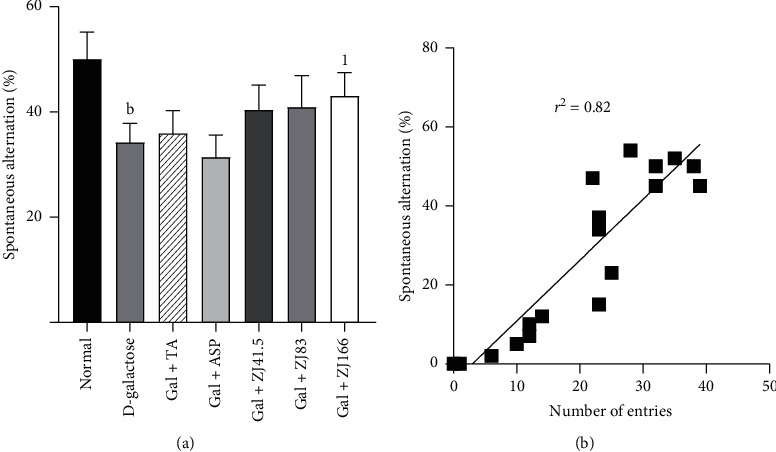
Effect of *Z. jujuba* extract on spontaneous alternation. (a) Spontaneous alternation behavior. (b) Correlation between spontaneous alternation and the number of arm entries. Each value represents the mean ± SEM; *n* = 7; b*P*<0.01: significant difference compared to the normal group; 1*P*<0.05: significant difference compared to D-galactose group; DW: distilled water; Gal: D-galactose; ASP: aspirin; TA: tacrine; ZJ: *Ziziphus jujuba*; Normal: normal group only treated with distilled water; D-galactose: negative control group treated with D-galactose and distilled water; Gal + TA: positive control group treated with D-galactose and tacrine (10 mg/kg); Gal + ASP: positive control treated with D-galactose and aspirin (20 mg/kg); Gal + ZJ (41.5, 83, and 166): test groups treated with D-galactose and the aqueous extract of *Z. jujuba* at the respective doses of 41.5, 83, and 166 mg/kg; *r*^2^: Correlation coefficient.

Administration of D-galactose to the D-galactose group reduced (*P*<0.01) the spontaneous alternation percentage compared to the normal control group (Figure 3(a)). Only the extract at a dose of 166 mg/kg increased (*P*<0.05) this percentage (Figure 3(a)) when tacrine or aspirin failed to do it (Figure 3(a)).

The linear regression curve showed that there is a strong correlation (*r*^2^ = 0.82) between spontaneous alternation behavior and the number of arm entries (Figure 3(b)).

### 3.3. Effect of *Z. jujuba* on the Relative Weight of the Prefrontal Cortex

Administration of D-galactose to the D-galactose group increased the mass of the prefrontal cortex to 12.80% (*P*<0.05) compared to the normal group (Table 1). The extract at doses of 41.5 and 166 mg/kg reduced this mass to 6.40% (*P*<0.05). Tacrine, as well as aspirin, reduced also this mass to 7.40% (*P* < 0.05) and 7.20% (*P* < 0.05), respectively (Table 1).

Each value represents the mean ± SEM; *n* = 7; a*P*<0.05: significant difference compared to the normal group; 1*P*<0.05: significant difference compared to D-galactose group; DW: distilled water; Gal: D-galactose; ASP: aspirin; TA: tacrine; ZJ: *Ziziphus jujuba*; Normal: normal group treated only with distilled water; D-galactose: negative control group treated with D-galactose and distilled water; Gal + TA: positive control group treated with D-galactose and tacrine (10 mg/kg); Gal + ASP: positive control treated with D-galactose and aspirin (20 mg/kg); Gal + ZJ (41.5, 83, and 166): test groups treated with D-galactose and the aqueous extract of Z*. jujuba* at the respective doses of 41.5, 83, and 166 mg/kg.

### 3.4. Effects of *Z. jujuba* on the Cholinergic Parameters

**Figure 4:**
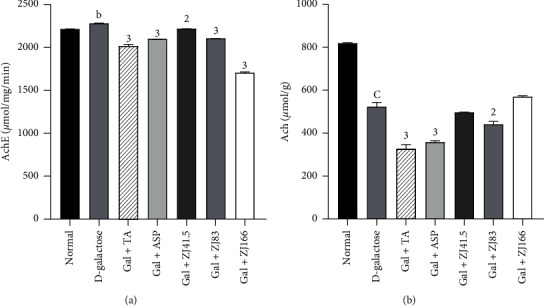
Effect of *Z. jujuba* extract on the cholinergic parameters. (a) AchE activity. (b) Ach level. Each value represents the mean ± SEM; *n* = 5; b*P*<0.01, c*P*<0.001: significant difference compared to the normal group; 2*P*<0.01, *P*<0.001: significant difference compared to D-galactose group; DW: distilled water; Gal: D-galactose; ASP: aspirin; TA: tacrine; ZJ: *Ziziphus jujuba;* Normal: normal group only treated with distilled water; D-galactose: negative control group treated with D-galactose and distilled water; Gal + TA: positive control group treated with D-galactose and tacrine (10 mg/kg); Gal + ASP: positive control treated with D-galactose and aspirin (20 mg/kg); Gal + ZJ (41.5, 83, and 166): test groups treated with D-galactose and the aqueous extract of *Z. jujuba* at the respective doses of 41.5, 83, and 166 mg/kg; AchE; acetylcholinesterase; Ach: acetylcholine.

Administration of D-galactose to the D-galactose group increased the activity of AchE to 2282.30 ± 8.92 *μ*mol/mg/min (*P*<0.01) compared to the normal group (Figure 4(a)). The extract at doses of 41.5, 83, and 166 mg/kg markedly reduced this activity to 2% (*P*<0.001), 7% (*P*<0.001), and 25% (*P*<0.001), respectively. Tacrine and aspirin decreased this activity to 11.6% (*P*<0.001) and 7.9% (*P*<0.001) (Figure 4(a)).

In the prefrontal cortex, the level of Ach decreased to 524.69 ± 2.23 *μ*mol/g (*P*<0.001) in the negative control group compared to the normal group (Figure 4(b)). The extract at the dose of 83 mg/kg, as well as tacrine or aspirin, reduced (*P*<0.01) the Ach level to 441.33 ± 16.71 (*P*<0.01), 325.93 ± 23.40 (*P*<0.001), and 358.75 ± 9.20 (*P*<0.001), respectively (Figure 4(b)).

### 3.5. Effects of *Z. jujuba* on Glial Cells Activation Markers

**Figure 5:**
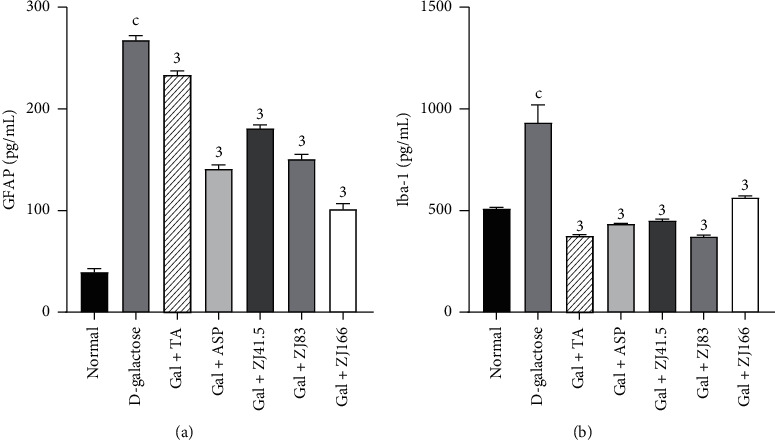
Effect of *Z. jujuba* extract on glial cells activation markers. (a) GFAP level. (b) Iba-1 level. Each value represents the mean ± SEM; *n* = 5; c*P*<0.001: significant difference compared to the normal group; 3*P*<0.001: significant difference compared to D-galactose group; DW: distilled water; Gal: D-galactose; ASP: aspirin; TA: tacrine; ZJ: *Ziziphus jujuba;* Normal: normal group only treated with distilled water; D-galactose: negative control group treated D-galactose and distilled water; Gal + TA: positive control group treated with D-galactose and tacrine (10 mg/kg); Gal + ASP: positive control treated with D-galactose and aspirin (20 mg/kg); Gal + ZJ (41.5, 83, and 166): test groups treated with the D-galactose and the aqueous extract of *Z. jujuba* at the respective doses of 41.5, 83, and 166 mg/kg; GFAP; glial fibrillary acidic protein; Iba-1: ionized calcium-binding adaptor molecule 1.

In the D-galactose group, the level of GFAP increased to 267.54 ± 4.85 pg/mL (*P*<0.001) compared to the normal group (Figure 5(a)). However, the highest dose of the extract showed the greatest effect by reducing this level to 62.22% (*P*<0.001). Tacrine and aspirin decreased this parameter to 12.70% (*P*<0.001) and 47.3% (*P*<0.001), respectively (Figure 5(a)).

In the prefrontal cortex, the concentration of Iba-1 increased to 933.5 ± 8.40 pg/mL (*P*<0.001) in the negative control group compared to the normal group (Figure 5(b)). However, the extract (83 mg/kg) showed the most effective effect by reducing this level to 60% (*P*<0.001). Tacrine and aspirin equally decreased (*P*<0.001) this parameter to 59.80% (*P*<0.001) and 53.48% (*P*<0.001), respectively (Figure 5(b)).

### 3.6. Effects of *Z. jujuba* on Some Proinflammatory Cytokines

**Figure 6:**
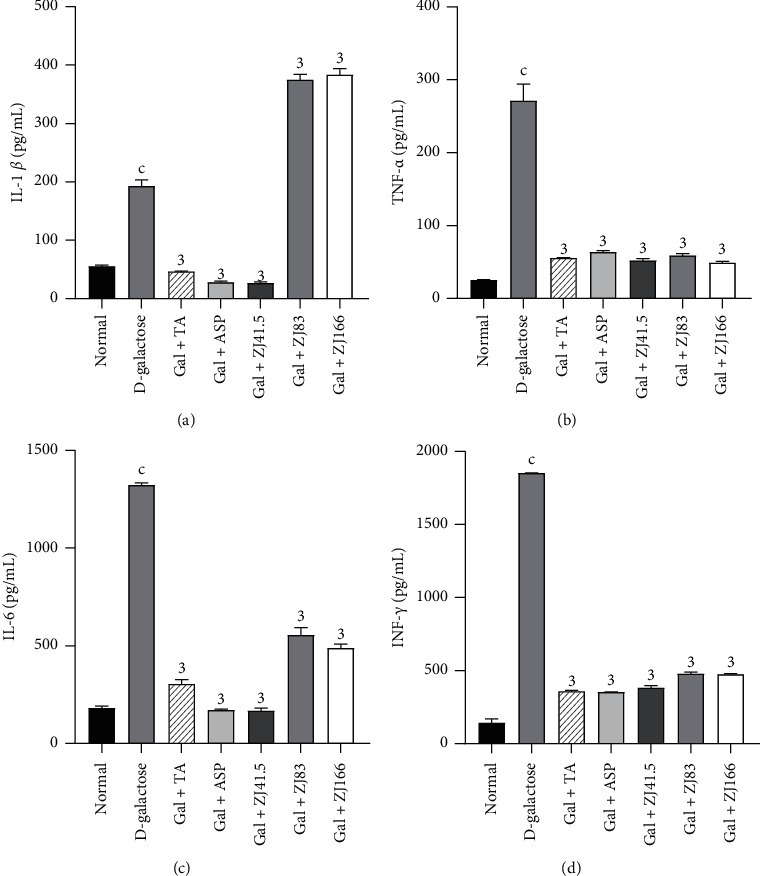
Effect of *Z. jujuba* extract on some proinflammatory cytokines. (a) IL-*β* level, (b) TNF-*α* level, (c) IL-6 level, and (d) INF-*γ* level. Each value represents the mean ± SEM; *n* = 5; c*P*<0.001: significant difference compared to the normal group; 3*P*<0.001: significant difference compared to D-galactose group; DW: distilled water; Gal: D-galactose; ASP: aspirin; TA: tacrine; ZJ: *Ziziphus jujuba*; Normal: normal group only treated with distilled water; D-galactose: negative control group treated with D-galactose and distilled water; Gal + TA: positive control group treated with D-galactose and tacrine (10 mg/kg); Gal + ASP: positive control treated with D-galactose and aspirin (20 mg/kg); Gal + ZJ (41.5, 83, and 166): test groups treated with D-galactose and the aqueous extract of *Z. jujuba* at the respective doses of 41.5, 83, and 166 mg/kg; TNF-*α*, tumor necrosis factor-alpha; IL-*β*, interleukin beta; IL-6: interleukin 6; INF-*γ*, interferon-gamma.

The level of IL-1*β* increased to 193.18 ± 11.24 pg/mL in the D-galactose group compared to the normal group (Figure 6(a)). The extract at a dose of 41.5 mg/kg optimally decreased (*P*<0.001) this level to 86.12% (*P*<0.001). Tacrine and aspirin also decreased this level to 75.64% (*P*<0.001) and 85.49% (*P*<0.001), respectively (Figure 6(a)).

The level of TNF-*α* increased to 271.56 ± 10.04 pg/mL (*P*<0.001) in the D-galactose group compared to the normal group (Figure 6(b)). The extract (41.5, 83, and 166 mg/kg) decreased this level to 80.81% (*P*<0.001), 78.22% (*P*<0.001), and 81.89% (*P*<0.001), respectively. Similarly, tacrine and aspirin reduced this level to 79.42% (*P*<0.001) and 76.41% (*P*<0.001), respectively (Figure 6(b)).

Administration of D-galactose increased the level of IL-6 to 1323.29 ± 15.32 pg/mL (*P*<0.001) in the D-galactose group compared to the normal group (Figure 6(c)). The extract at a dose of 41.5 mg/kg optimally decreased this level to 87.33% (*P*<0.001). Tacrine and aspirin also reduced this level to 76.94% (*P*<0.001) and 86.98% (*P*<0.001), respectively (Figure 6(c)).

The level of INF-*γ* increased to 1853.30 ± 3.22 pg/mL (*P*<0.001) in the D-galactose group compared to the normal group (Figure 6(d)). The extract at doses of 41.5, 83, and 166 mg/kg reduced this level to 79.38% (*P*<0.001), 74.10% (*P*<0.001), and 74.36% (*P*<0.001), respectively. Tacrine and aspirin similarly decreased this parameter to 80.62% (*P*<0.001) and 80.90% (*P*<0.001), respectively (Figure 6(d)).

### 3.7. Effect of Z. *jujuba* Extract on the Neuronal Alterations in the Prefrontal Cortex

**Figure 7:**
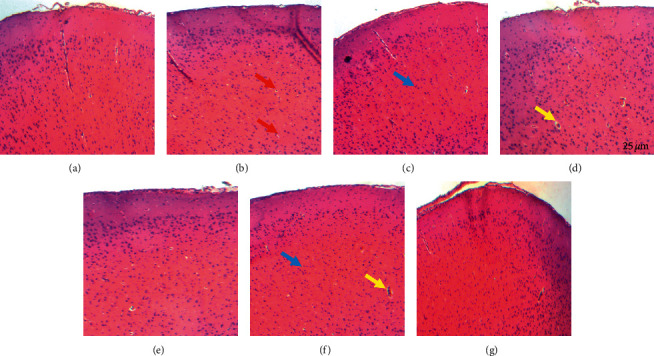
Photomicrographs of the prefrontal cortex sections after hematoxylin and eosin staining (×250). (a) Normal control group; (b) D-galactose group; (c) positive control treated with tacrine (10 mg/kg); (d) positive control treated with aspirin (20 mg/kg); (e) group treated with *Z. jujuba* (41.5 mg/kg); (f) group treated with *Z. jujuba* (83 mg/kg); (g) group treated with *Z. jujuba* (166 mg/kg). Normal neuron (blue arrow); perivascular edema and dilated blood vessel (yellow arrow); granulovacuolar degeneration and pyknosis (red arrow).

Normal neuronal density was observed in the prefrontal cortex of the normal control group (Figure 7(a)). In the D-galactose group, granulovacuolar degeneration and reduced neuronal density were observed (Figure 7(b)). Perivascular edema was also observed in some neurons of the prefrontal cortex (Figure 7(b)). Rats treated with the extract (166 mg/kg) had normal neuronal density (Figure 7(g)). In the group of rats treated with tacrine or aspirin, reduced neuronal density and granulovacuolar degeneration were observed (Figures 7(c) and 7(d).

### 3.8. Qualitative Phytochemical Analysis

Phytochemical screening revealed that the extract contains traces of triterpenes and anthraquinones, small quantities of alkaloids and saponins, and large amounts of flavonoids, tannins, sterols, and glycosides (Table 2).

### 3.9. Quantitative Phytochemical Analysis

Results showed that when compared to standards, the total phenolic compounds (124.33 ± 0.12 mg GAE/g), condensed tannins (15.22 ± 0.60 mg CatE/g) flavonoids contents (14.09 ± 0.45 mg RE/g), and total alkaloids (20.09 ± 0.12%) were abundant, while saponins (9.89 ± 0.23%) were less abundant (Table 3).

## 4. Discussion

This study aimed to determine the antiamnesic and anti-inflammatory effects of *Ziziphus jujuba* aqueous extract in D-galactose-induced working memory impairment in rats.

Loss of memory is a leading symptom often reported by patients suffering from Alzheimer's disease (AD), and working memory is affected early on in the progression of the disease [39, 40]. To induce memory disorders in rodents, many experimental models are used, including the D-galactose model. The D-galactose model is a well-known model for studying aging and related oxidative damage and memory impairment [50, 51]. Indeed, chronic D-galactose administration in rodents induces Alzheimer's-like cognitive deficits, resulting in impaired short or long-term memory [50, 51]. In the present study, the subcutaneous injection of D-galactose in rats resulted in a significant increase in the time taken to explore the old object over the new (novel) object and a decrease in the discrimination index in rats. These observations were confirmed by a decrease in the spontaneous alternation behavior and corroborate the large body of evidence on D-galactose-induced working memory impairment in rodents [50, 52]. D-galactose is reducing sugar in the body that is metabolized by D-galactokinase and galactose-1-phosphate uridyltransferase in animals. However, elevated levels of D-galactose result in metabolic abnormalities [53]. D-galactose is converted into galactitol, which is not metabolized but accumulates in the cell, leading to osmotic stress and production of reactive oxygen species, resulting in neuroinflammation, neurodegeneration, and memory impairment [53]. Administering *Ziziphus jujuba* extract to mice treated with D-galactose improved the time taken to explore the new object and the spontaneous alternation, suggesting a protective effect on the working memory deficit [54]. Similar results on the antiamnesic effect of the ethanolic extract of *Z. jujuba* on cognitive dysfunctions support this hypothesis [25]. The protective effect of *Z. jujuba* fruit extract on spatial memory impairments corroborates the antiamnesic effect of *Z. jujuba* extract [26] and justifies the fact that different parts of a plant may possess identical pharmacological properties. Also, the effect of the extract was better than that of tacrine, an effective anticholinesterase agent against Alzheimer's disease, whose mechanism of action is to inhibit the metabolism of acetylcholine by preventing the degradation of acetylcholine by the AchE [55]. So, these findings also suggest an interference with the metabolism of acetylcholine, possibly by the inhibition of the AchE activity, and other mechanisms which need to be explored. Furthermore, flavonoids and alkaloids have been reported to prevent memory impairment in rats via the inhibition of AchE, inflammation, and oxidative stress [56]. Given that flavonoids and alkaloids were abundantly found in the extract, these findings point out their role in the antiamnesic effect of the extract.

Accumulating evidence showed that memory loss is often associated with the atrophy of the prefrontal cortex (PFC). Therefore, analyzing the relative weight of the PFC may give insight into the mechanism (s) through which the extract exhibits its antiamnesic effect [45]. The PFC is a heterogeneous cortical structure that supports mainly cognitive functions, including working memory and verbal abilities [57]. Consequently, its atrophy is highly associated with dementia and can be considered as a predictor of AD [58]. In the present study, administration of D-galactose in distilled water-treated rats decreased the mass of the prefrontal cortex. This observation corroborates the evidence on the PFC neurodegeneration in healthy aging and AD patients. Indeed, in subjects with AD, both gray and white matter loss contribute to the reduction of PFC volume [59]. In contrast, the administration of the extract (41.5 and 166 mg/kg), as well as tacrine or aspirin, moderately reduced the atrophy of the prefrontal cortex when compared to the D-galactose group, suggesting a neuroprotective effect against neuronal loss in the prefrontal cortex. Indeed, it has been demonstrated that the atrophy of the PFC is related to neuronal loss, including cholinergic neurons involved in memory processes [59]. These results explain in part the antiamnesic effect of the extract against D-galactose-induced memory impairment [60]. The presence in the extract of flavonoids, alkaloids, and phenols with potent neuroprotective activity may also explain the protective effect of the extract in the present study [61].

In AD patients, the activity of AchE is significantly decreased [62]. Similar patterns were found in aged rodents [62]. AchE causes the degradation of Ach which produces cholinergic deficits. The inhibition of this enzyme is considered the best strategy to treat AD and dementia [63, 64]. The administration of D-galactose increased the activity of AchE and decreased the level of Ach [64]. In the animal model of AD, it has been shown that chronic administration of D-galactose leads to AchE activity alteration and death of cholinergic neurons [42, 43]. Besides, it is well known that the administration of D-galactose triggers oxidative stress, resulting in cholinergic alterations through increased AchE activity and decreased Ach levels [63, 64]. According to accumulating evidence on the dysfunctions of the cholinergic pathway, oxidative stress and neuroinflammation are mainly involved [25, 64]. The administration of the extract of *Z. jujuba* (83 and 166 mg/kg) resulted in the inhibition of the AchE activity and the increase of Ach level. This activity of the extract was greater than that of tacrine, an irreversible cholinesterase inhibitor, and first-line agents in the management of cognitive impairments. Since Ach is the main neurotransmitter involved in the memory process, these data explain the antiamnesic effect of the extract likely through the inhibition of the AchE activity [25, 63]. Moreover, the extract improved the level of Ach compared to the control group, and these results also indicate a nootropic effect of the extract [34]. Several studies have shown that flavonoids and polyphenols inhibited AchE activity and increased Ach level; given that these compounds are abundant in the extract, these findings suggest their role in cholinergic modulation [25, 63].

Among possible mechanisms involved in cognitive impairments, inflammation is mainly cited [65–67]. Inflammation is a biological process adapted in response to many stimuli. Although beneficial in some cases, proinflammatory cytokine production may have deleterious effects in exaggerated responses [65–67]. The chronic inflammatory process contributes to worsening brain injuries. In AD, the main engine of activation of microglia cells and astrocytes is the presence of A*β*P [65–67]. Also, exposure of microglia to preaggregated A*β*1-42 increases the production of proinflammatory cytokines (i.e., IL-1*β*, IL-6, and TNF-*α*), macrophage inflammatory peptide, and macrophage colony-stimulating factor [46–48]. The increased deposition of A*β*P stimulates the inflammation process, which in turn leads to neuronal dysfunction by three main mechanisms: production of cellular (GFAP and Iba-1) and molecular proinflammatory markers (IL-1*β*, TNF-*α*, IL-6, and INF-*γ*) [65–67]. Therefore, preventing or altering neuroinflammation could constitute an effective therapeutic strategy to cure AD. The administration of D-galactose increased the level of proinflammatory markers. These data are consistent with those of Lei et al. [68] on cellular proinflammatory markers (GFAP and Iba-1) and those of Zhang et al. [64] on molecular proinflammatory markers (IL-1*β*, TNF-*α*, IL-6, and INF-*γ*). The extract reversed all these effects, suggesting an anti-inflammatory activity [69]. Moreover, aspirin, which is a nonsteroid anti-inflammatory drug (NSAIDs), inhibits the activity of the enzyme called cyclooxygenase (COX). This action triggers the formation of prostaglandins (PGs), resulting in inflammation, swelling, pain, and fever [70]. Besides, it has been reported that aspirin at a high dose lowers the prevalence of Alzheimer's dementia and had better cognitive function than nonusers [71]. One of the mechanisms by which aspirin alleviates dementia in AD includes the inhibition of glutamate release [72]. Given that the effect of the extract was more marked than that of aspirin, these results indicate that the anti-inflammatory activity of the extract passes through the inhibition of COX activity and other anti-inflammatory pathways. Complementary studies need to be done to determine the exact anti-inflammatory mechanisms of the extract. The anti-inflammatory activity of the extract might be explained by the presence of secondary metabolites with anti-inflammatory potential. Indeed, flavonoids, alkaloids, and terpenes have been shown to induce anti-inflammatory effects through the trapping of kinases (PKC, PI3kinase, NFkB, and tyrosine kinases), as well as the inhibition of the expression of GFAP, Iba-1, and proinflammatory cytokines [73, 74]. These findings point to the fact that the antiamnesic effect of the extract is mediated in part by inhibiting chronic inflammation.

The analysis of the histological sections of the PFC of the galactose-treated rats showed a reduction in the number of neuronal cells in the prefrontal cortex. These data are in agreement with those of Rahman et al. [75] on the effects of anthocyanins on D-galactose-induced oxidative stress and neuroinflammation and cognitive impairment in adult rats. The extract protected the prefrontal cortex against neuronal injury induced by D-galactose. This protection was reflected by a preserved density of neurons and intact neurons when compared to D-galactose-treated rats. Assuming that the prefrontal cortex is mainly involved in the working memory process [75, 76], these findings confirm the neuroprotective effect of the extract as hypothesized above. This effect seems to be mediated in part by anti-inflammatory activities when compared to aspirin, an anti-inflammatory drug, where its mechanism of action includes the inhibition of apoptosis signaling pathway [54, 77]. Recently, studies have revealed that polyphenolic compounds, including flavonoids, phenolic acids, alkaloids, carotenoids, catechins, and terpenes, have great potential in treating neurodegenerative diseases [78]. Since polyphenols are the most abundant compound in the extract, these constituents might account for the neuroprotective effect of this extract. Other studies on the neuroprotective effect of the extract using immunohistochemistry techniques must be done to confirm the obtained results. Altogether, the antiamnesic effect of the extract of *Z. jujuba* appears to be mediated in part by cholinergic and anti-inflammatory modulations. This antiamnesic effect of the extract was more marked than that of tacrine or aspirin. Thus, this extract, which combines antiamnesic and anti-inflammatory effects, could be a candidate for the development of effective and safer drugs against AD. Further studies on isolated bioactive molecules from the extract could help establish the exact mechanisms involved in the antiamnesic and anti-inflammatory effects of the extract.

## 5. Conclusions

Treatment with the aqueous extract protected the animals from the harmful effects of D-galactose on working memory. Indeed, the extract significantly improved the working memory in the object recognition and Y-maze tests, suggesting, therefore, an antiamnesic effect. It also increased the bioavailability of Ach and reduced the level of proinflammatory cytokines. These results indicate the involvement of cholinergic and anti-inflammatory pathways in the antiamnesic effect of the extract. These findings justify the use of this herb in the treatment of dementia and other neurological disorders in Cameroon's alternative medicine. Furthermore, this extract could be used as an adjunct in the prevention of dementia in AD patients.

## Figures and Tables

**Table 1:** Effect of *Z. jujuba* extract on the relative weight of prefrontal cortex.

Treatment	Relative weight (%)
Normal	7.40 ± 0.08
D-galactose	12.80 ± 0.01a
Gal + TA	7.40 ± 0.011
Gal + ASP	7.20 ± 0.081
Gal + ZJ41.5	6.40 ± 0.011
Gal + ZJ83	9.00 ± 0.01
Gal + ZJ166	6.40 ± 0.011

**Table 2:** Phytochemical analysis of the aqueous extract of *Z. jujuba*.

Chemical compound	Result
Alkaloids	++
Flavonoids	+++
Tannins	+++
Anthraquinones	+
Sterols and polyphenols	+++
Saponins	++
Glycosides	+++
Triterpenes	+

+ = weakly positive, ++ = moderately positive, +++ = strongly positive.

**Table 3:** Quantitative analysis of the aqueous extract of *Z. jujuba*.

Chemical compounds	Unit	Mean
Alkaloids	%	20.09 ± 0.12
Flavonoids	mg RE/g	14.09 ± 0.45
Tannins	mg CatE/g	15.22 ± 0.60
Polyphenols	mg GAE/g	124.33 ± 0.12
Saponins	%	9.89 ± 0.23

GAE: gallic acid equivalent; CatE: catechin equivalent; RE: rutin equivalent; %: percentage.

## Data Availability

The data used to support the findings of this study are available from the corresponding author upon request.

## Abbreviations

ANOVA: Analysis of variance
DW: Distilled water
i.p.: Intraperitoneally
*p.o.*: *Per os*
s.c.: Subcutaneously
*Z. jujuba*: *Ziziphus jujuba*
Gal: D-galactose
SEM: Standard error of the mean
TNF-*α*: Tumor necrosis factor-alpha
IL-1*β*: Interleukin 1 beta
IL-6: Interleukin 6
INF-*γ*: Interferon-gamma
GFAP: Glial fibrillary acidic protein
Iba-1: Ionized calcium-binding adaptor molecule 1
AchE: Acetylcholinesterase
Ach: Acetylcholine
AD: Alzheimer's disease
DI: Discrimination index.
